# Association between antimicrobial usage and resistance in *Salmonella* from poultry farms in Nigeria

**DOI:** 10.1186/s12917-021-02938-2

**Published:** 2021-07-02

**Authors:** Abdurrahman Hassan Jibril, Iruka N. Okeke, Anders Dalsgaard, John Elmerdahl Olsen

**Affiliations:** 1 Department of Veterinary and Animal Sciences, Faculty of Health and Medical Sciences, University of Copenhagen, Copenhagen, Denmark; 2 Department of Veterinary Public Health and Preventive Medicine, Faculty of Veterinary Medicine, Usmanu Danfodiyo University Sokoto, Sokoto, Nigeria; 3 Department of Pharmaceutical Microbiology, Faculty of Pharmacy, University of Ibadan, Ibadan, Nigeria; 4 School of Chemical and Biomedical Engineering, Nanyang Technological University, Singapore, Singapore

**Keywords:** Antimicrobial usage, Antimicrobial resistance, *Salmonella*, Poultry, Nigeria

## Abstract

**Background:**

Antimicrobial resistance (AMR) is a global health threat affecting treatment outcome in animals and humans. A pre-requisite for development of AMR reduction strategies is knowledge of antimicrobial use patterns, and how these affect resistance development. The aim of this study was to determine antimicrobial usage (AMU) and whether such usage was associated with AMR in *Salmonella* from poultry farms in Northwest Nigeria.

**Results:**

Fifteen (37%) of antimicrobial products observed contained compounds that are of highest priority and critically important for human medicine. Broilers chicken consumed higher (28 ± 14 mg/kg active ingredients) amounts of antimicrobials compared to layers (13 ± 8 mg/kg) per week (*p* = 0.0009). Surprisingly, chickens raised under backyard system consumed higher amounts of antimicrobials (34 ± 7 mg/kg) than poultry in other systems (*p* = 0.02). High levels of resistance to tetracycline (58%), sulphonamides (65%), ciprofloxacin (46%) and gentamicin (42%) correlated with high farm level usage of these antimicrobials, and there was a strong correlation (r = 0.9) between farm usage and resistance of isolates to the same antimicrobials (*p* = 0.03).

**Conclusion:**

High AMU, including use of highest priority critically important antimicrobials was observed at poultry farms in Northwest Nigeria. AMU correlated with high levels of resistance. Communication of prudent use of antimicrobials to farmers and regulation to obtain reduction in AMU should be a priority.

**Supplementary Information:**

The online version contains supplementary material available at 10.1186/s12917-021-02938-2.

## Background

In 2015, the World Health Organization (WHO) launched its Global Action Plan on antimicrobial resistance (AMR) with one of its key objectives being the development and enhancement of monitoring systems for antimicrobial usage (AMU) worldwide [1]. The World Organization for Animal Health (OIE) also recognises that antimicrobial resistance is a global public and animal health concern that is influenced by the use of antimicrobial agents in both humans and animals, and it is suggested that OIE member-countries adopt international standards on the prudent use of antimicrobial agents and monitor antimicrobial usage in food producing animals [2]. However, measuring AMU in animal production in low- and middle-income countries (LMICs) is challenging due to the large numbers of small-scale farming units, access to antimicrobials sold “over the counter,” and generally poorly enforced regulatory frameworks [3].

Antimicrobials are used in livestock production mainly to prevent and control diseases, but also as growth promoters and to protect animal welfare [4, 5]. Because of global increase in livestock production, AMU in animal production has been predicted to increase by 11.5% from 2017 to 2030 [6] mostly driven by increased animal protein consumption in LMICs [3].

AMU on farms selects for AMR bacteria and genetic determinants of resistance may spread to humans either through direct contact to livestock, consumption of contaminated foods or indirectly through environmental pathways, leading to reduced efficacy of treatment in both animals and humans [7, 8]. Since the initial observation on the potential link between veterinary use of antimicrobials (AMs) and AMR in bacteria [9], the link has been confirmed in several studies describing the association between AMU and AMR, using combined AMU/AMR surveillance data [10–12].

As part of the recommendation by OIE, studies targeted to evaluate antimicrobial usage in food animals should include information about the usage pattern by species of animal and antimicrobials used in farms for a specific period of time [2]. In Nigeria and neighbouring Cameroun, high usage of antimicrobials has been reported in poultry farms by qualitative assessments using structured questionnaires [13, 14], but these data were not evaluated and correlated to AMR levels, and the validity of the AMU data was not confirmed by real observations on farms. The aim of the present study was to investigate antimicrobial usage in poultry farms in Nigeria, and to determine how such usage correlated with data on AMR in *Salmonella* spp. isolated from the same farms.

## Results

### Types of antimicrobials used at farms

A total of 41 antimicrobial-containing products were observed on the farms. Of these, 36 products contained antimicrobial active ingredients (AAIs) only, while five also contained other substances such as vitamins. Twenty products contained two or more antimicrobials, 39 products were intended for oral use, and 34 products were in powdered form (Table 1).

**Table 1:** Characteristics of antimicrobial containing products observed in commercial poultry farms in Nigeria

Parameters	Products (%) (***n*** = 41)	Farms observed (%) (***n*** = 41)
*Product composition*		
Antimicrobial only	36 (88)	24 (59)
Antimicrobial with other substances	5 (12)	17 (41)
*Number of antimicrobial declared in products*		
1	15 (37)	5 (12)
2	20 (49)	13 (32)
3	2 (5)	11 (27)
4	4 (10)	12 (30)
*Route of administration*		
Oral in water	39 (95)	39 (99)
Parenteral	2 (5)	2 (5)
*Formulation*		
Injectable	2 (5)	2 (5)
Powder	34 (83)	21 (51)
Suspension	5 (12)	18 (44)

Twenty antimicrobial types were declared in the 41 antimicrobial preparations (Additional file 1). The most commonly-used antimicrobials were: doxycycline, oxytetracyclines, colistin and erythromycin which were used on 28 (68%), 22 (54%), 22 (54%) and 21 (51%) of farms, respectively.

The aminoglycosides neomycin, gentamicin and streptomycin were commonly used at 13 (32%), 15 (37%) and 15 (37%) of farms respectively. There was considerably lower usage of penicillin (2%) and furazolidone (2%) than other drugs (Table 2). Thirty-two (78.1%) of the products contained AAIs that are classified as critically important for human medicine i.e. erythromycin, colistin, quinolones, aminoglycosides, oxazolidone and penicillin. Among these, 15 (37%) products contained AAIs that are categorised as “Highest priority critically important” namely colistin and ciprofloxacin as listed by WHO [15] (Table 3).

**Table 2:** Antimicrobial classes and active substances administered to poultry in commercial farms in Nigeria

Antimicrobial class	Active ingredient	Farms observed (%) (***n*** = 41)
Aminoglycosides	Neomycin	13 (32)
	Gentamicin	15 (37)
	Streptomycin	15 (37)
Amphenicols	Florfenicol	6 (15)
Macrolides	Erythromycin	21 (51)
	Tylosin	15 (37)
Nitrofuran	Furazolidone	1 (2)
Penicillins	Amoxicillin	3 (7)
	Penicillin G	1 (2)
Polypeptides	Colistin	22 (54)
Pyrimidine derivatives	Amprolium	3 (7)
	Trimethoprim	8 (20)
Fluoroquinolones	Ciprofloxacin	1 (2)
	Enrofloxacin	13 (32)
Sulfonamides	Sulphadiazine	12 (30)
	Sulphadimidine	4 (10)
	Sulphathiazole	3 (7)
	Sulphaquinozalone	3 (7)
Tetracyclines	Doxycycline	28 (68)
	Oxytetracycline	22 (54)

**Table 3:** Highest priority and high priority antimicrobials observed in poultry farms in Nigeria

WHO classification	Number of products (***n*** = 41)	Percentage (%)
*Highest priority*	15	37
Macrolides	5	12
Polymyxins	6	15
Quinolones	4	10
Cephalosporins (3rd generation and above)	0	0
*High priority*	17	42
Aminoglycosides	13	32
Oxazolidone	1	2
Penicillin	3	7

### Quantification of antimicrobial usage

**Fig. 1:**
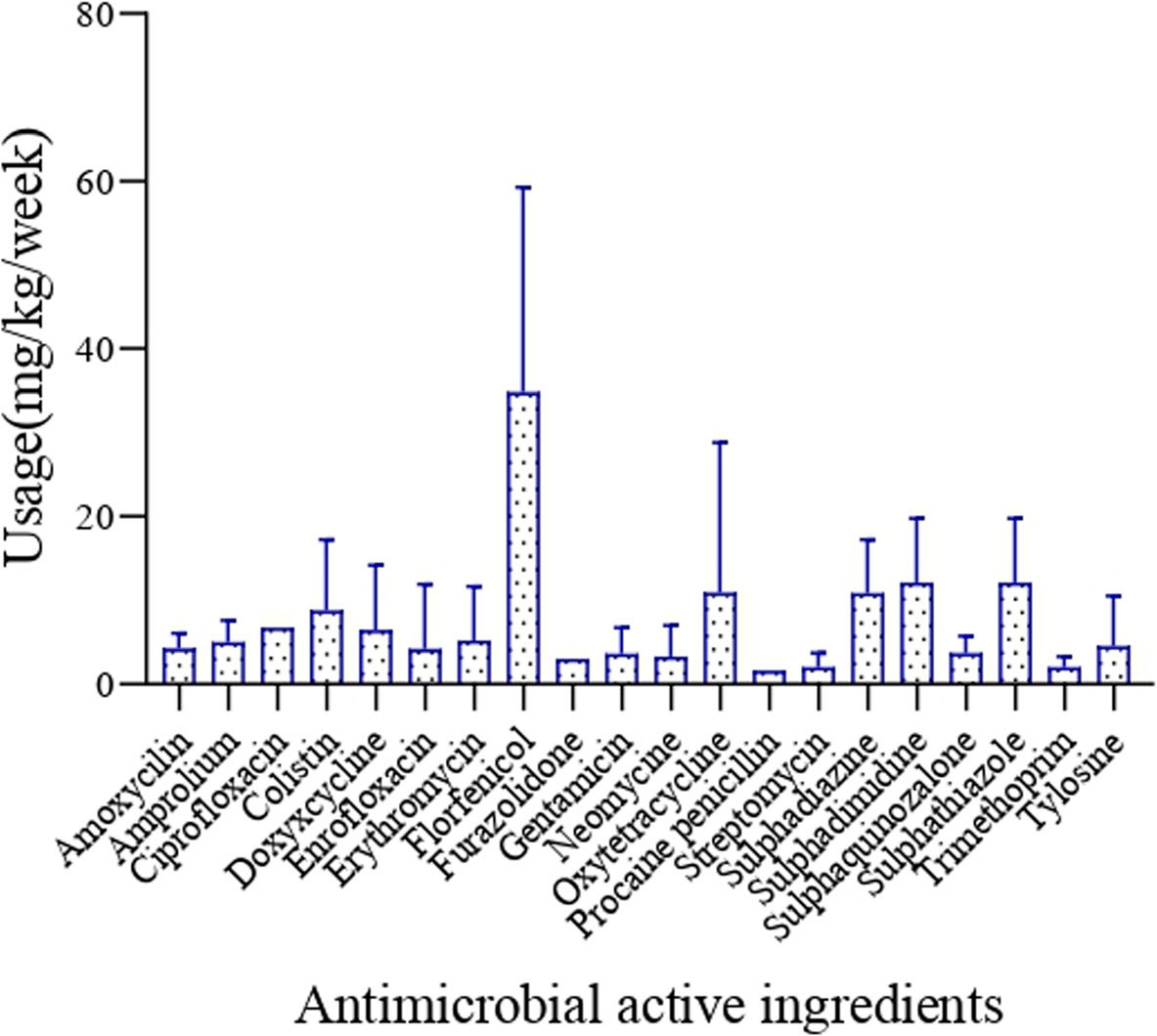
Average usage of antimicrobials (active ingredients in mg/kg per week) contained in 41 products obtained from 41 poultry farms in Northwest Nigeria

**Fig. 2:**
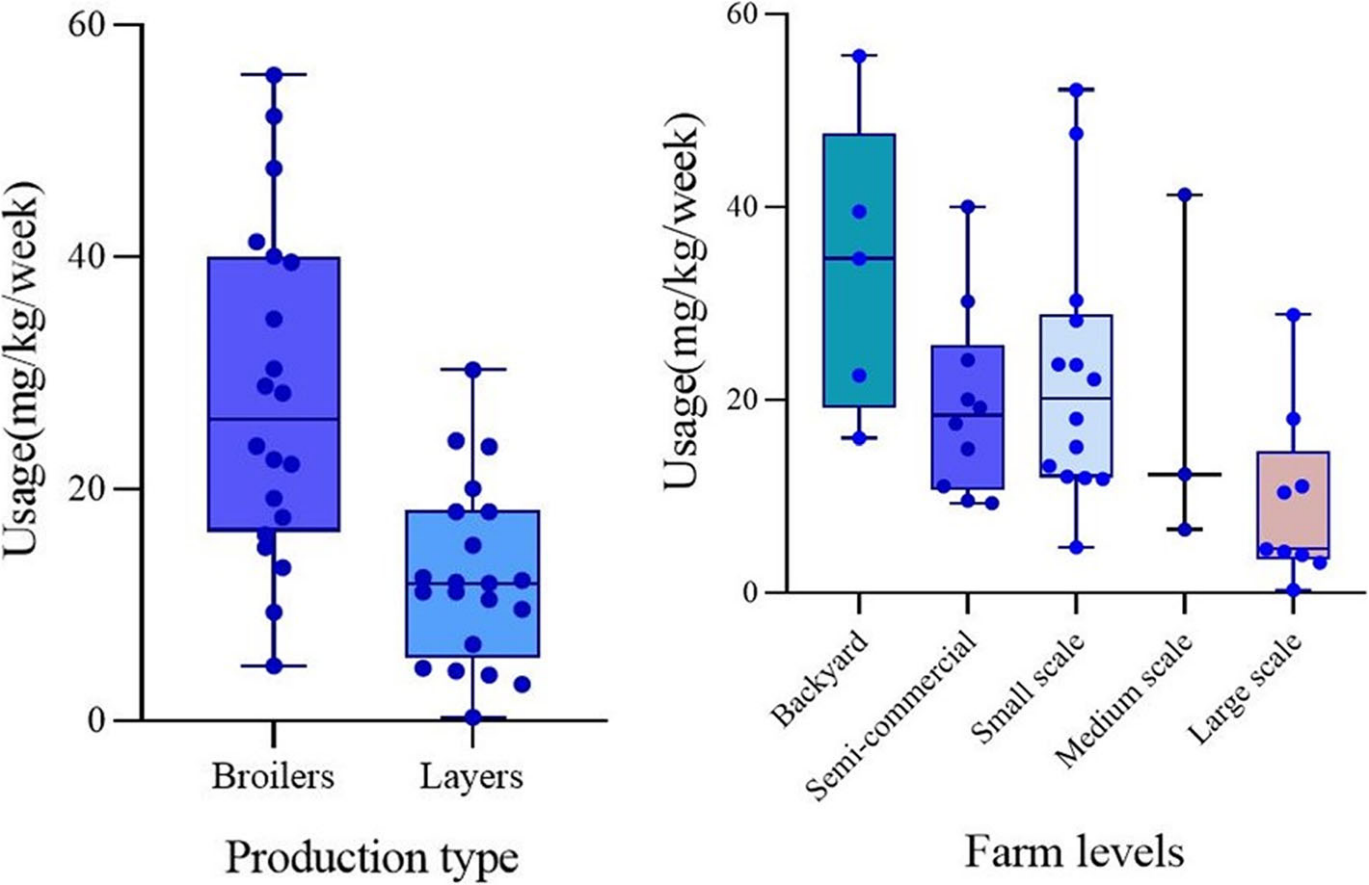
Left Hand Side: Variation (*p* = 0.0009) in quantity of antimicrobial usage in mg/kg per week based on production type. Right Hand Side: Variation (*p* = 0.02) in usage from the different categories of farms selected in the study area

Florfenicol had the highest average usage per chicken per week (15 mg), followed by sulphadimidine, sulphathiazole (5 mg each), oxytetracycline (5 mg), colistin (4 mg) and ciprofloxacin (3 mg), while tylosine, (2 mg), gentamicin (2 mg), neomycin (2 mg), trimethoprim (1 mg), and streptomycin (1 mg) were the ones used the less (Fig. 1). The estimated weight of broilers after the growth period of 6 weeks was 2.5 kg, while that of layers after 24 weeks was 1.8 kg. Based on this, broiler chickens consumed higher amounts of antimicrobial active ingredients (28 ± 14 mg/kg per week) than layers (13 ± 8 mg/kg per week) (*p* = 0.0009). Backyard chicken was found to consume more antimicrobials (34 ± 7 mg/kg per week) than chicken raised in other production systems (*p* = 0.02) (Fig. 2).

### Correlation between AMU and AMR

**Fig. 3:**
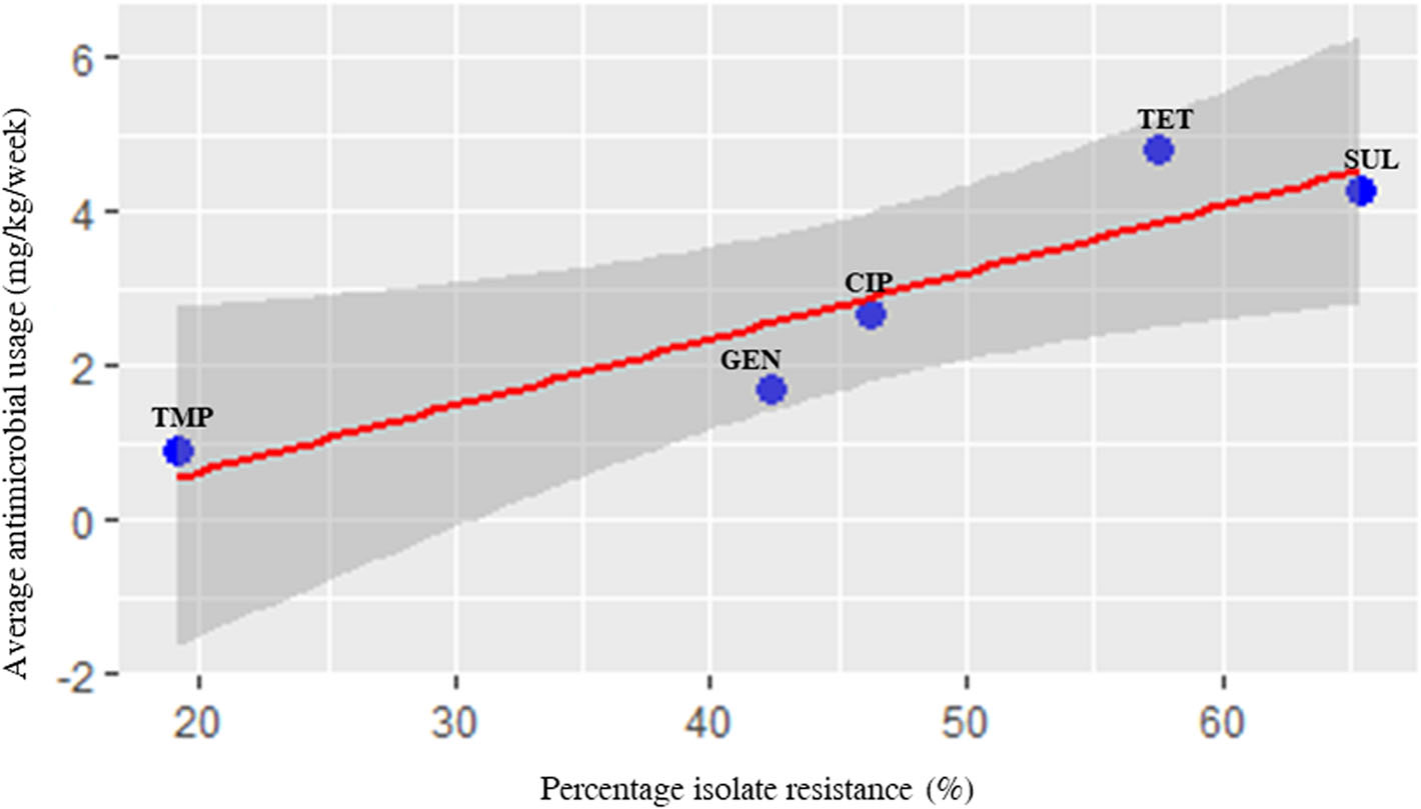
Scatter plot analysis indicating positive correlation between percentage of isolate resistance and average usage of active antimicrobial ingredients of selected antimicrobials. Each dot represents a specific antimicrobial. The blue colour represents the 95% confidence interval. CIP, ciprofloxacin; GEN, gentamicin; SUL, sulphonamides; TET, tetracycline; and TMP, trimethoprim

Antimicrobial resistance data for *Salmonella* isolated from the 41 farms were obtained from a previous study published by the same authors as this study [16]. Details of these isolates are shown in Additional file 2. There was a maximum of 30 and 120 days between *Salmonella* isolation and recordings of AMU use for broiler and layer farms, respectively. Five antimicrobial agents that were highly used in the farms and were part of the panel of antimicrobials used for susceptibility testing, were used to evaluate correlation between farm AMU and phenotypic resistance as well as  correlation between AMU and prevalence of antimicrobial resistance genes (ARGs) conferring resistance to these antimicrobials . The results showed that high level usage of tetracycline and sulphonamides correlated with high level of isolates showing resistance to these antibiotics. Likewise, moderate usage of gentamicin and ciprofloxacin correlated with moderate percentage of *Salmonella* isolates being resistant to the same antimicrobials (r = 0.9, [CI = 0.2–0.99], *p* value = 0.03), and low farm usage of trimethoprim correlated with a low number of isolates being resistant to this drug (Table 4). Similarly, there was strong correlation between usage and percentage of isolates that had resistant genes conferring resistance to the antimicrobials (r = 0.9, [CI = − 0.004-0.99] *p* = 0.05). However, this was not statistically significant. Scatter plot analysis of the resistance and usage variables also showed uphill trends, indicating a positive strong relationship between these two variables (*r* = 0.9, *p* = 0.03) (Fig. 3).

**Table 4:** Antimicrobial usage and resistance to selected antimicrobial agents used in commercial farms in Nigeria

Antimicrobials	Resistance (% of strains) (***n*** = 26)	Antimicrobial resistant genes present (% of strains) (***n*** = 26)	Average antimicrobial usage (mg/kg/week)	Correlation coefficients (r)
Trimethoprim	19	12	1	
Tetracycline	58	50	5	^a^{0.9 [CI = 0.2–0.99, *p* = 0.03]
Sulphonamides	65	46	4	^b^{0.83 [CI = −0.004-0.99, *p* = 0.05]
Gentamicin	42	35	2	
Ciprofloxacin	46	23	3	

Coefficient between antimicrobial usage and percent of isolates with resistance ^a^coefficient between antimicrobial usage and isolates with resistance genes ^b^, *n* Number of isolates, *C.I* Confidence interval.

Farms were categorized into high users and low users of antimicrobials (see Materials and Methods section), and data was used as inputs in modelling risk factors for presence of AMR in *Salmonella* at the farm. From the univariate analysis, magnitude of AMU and farm category was observed to have statistical significance on the resistance level of *Salmonella* isolates, while production type (broilers vs. layers) showed no significance, and this factor was therefore excluded in the logistic regression analysis. This observation made sense, since the interpretation was that high use of antimicrobials had the same effect in the two types of productions. In the second step, logistic regression analysis was used to evaluate if the magnitude of AMU could predict isolate resistance at farm level. From the logit equation, the results indicated that no AMU was associated with little resistance with a log odds of resistance equal − 0.4. There was a significant difference in the log odds of AMR by 2.8 between farms with low and high AMU. With the exception of large-scale farms, the log odds of AMR increased with an increase in flock size, however, the difference was not found to be statistically significant (Table 5).

**Table 5:** Logistic regression analysis between antimicrobial susceptibility of isolates and magnitude of antimicrobial usage with farm as random effect

Predictors	Estimate	± SE	***p*** value
Intercept	−0.4	0.9	0.66
**Usage**			
High	reference		
Low	−2.8	1.3	0.03
**Farm category**			
Backyard	reference		
Large scale	−0.5	1.5	0.73
Medium scale	2.2	1.9	0.23
Semi-commercial	1.9	1.6	0.13
Small-scale	1.2	1.1	0.28

Production type (broiler and layers) was excluded in the logistic regression analysis, due to lack of significance with antimicrobial susceptibility of isolates in the first step univariate analysis.

## Discussion

This study assessed type of antimicrobials used in poultry farms in Northwest Nigeria, quantified antimicrobial usage (AMU) in all categories of commercial poultry farms, and furthermore analysed for associations between AMU and levels of resistance (AMR) in *Salmonella* isolated in those farms.

Oral treatment was the most frequently used route of antimicrobial administration in poultry. About 95.1% of the products were oral formulations, and in 98.1% of the farms visited, the preferred method for administration was mass medication via drinking water. This finding is in agreement with a previous study that reported 80% of drugs were administered in drinking water in poultry raised in the Abia State state of Nigeria [17]. Drug administration via feed was not used likely because it is difficult for farmers to add and mix antimicrobials into feed, but also because sick chickens will continue to drink, but have a lower feed intake [14]. Oral medication via water is the most preferred route of drug administration of poultry, purposely because whole flocks can be treated over long periods. Consequently, large number of chickens, and their proximal environment, will experience long-term exposure to many different classes of antimicrobials exerting a high pressure for selection of bacterial resistance. This is likely to lead to increased levels of resistance compared to treatment of smaller groups, however, this need to be further investigated. Surprisingly, single animal or small group treatment was reported to have no significant influence on antimicrobial resistance development in nursery pigs [18], probably because treated and untreated pigs mingle, and a similar situation may exist in poultry farm. A further problem linked to mass medication via water is the likelihood of inadvertent under dosing due to reduced bioavailability, e.g. by inhomogeneous mixtures, chemical degradation of the drug, adverse interactions with feed components, or other drugs, and reduced feed or water intake by the diseased animals [5]. Individual therapy with injectable antimicrobials was only reported in 4.9% of farms and was mostly administered by large-scale farms that adhered to a principle of treatment of sick chickens in an isolation pen.

Florfenicol, oxytetracycline, sulphonamides ciprofloxacin and gentamicin were the most frequently used antimicrobials. This finding corroborates recent findings from a qualitative survey conducted by Al-Mustapha et al. [19] that observed gentamicin, sulfonamide and quinolone-based antimicrobials as the most frequently administered antimicrobials in poultry in Kwara State, Nigeria. Another study reported that clotrimoxazole, neomycin, oxytetracycline and chloramphenicol were the most frequently used antimicrobials in Ile-Ife State, Nigeria [20]. With the exception of florfenicol, all the antimicrobials used in the sampled poultry farms belong to the same classes of antimicrobials used in human medicine. It is particularly worrisome that many products contained AAIs that are categorised as “Highest priority critically important” for human health as listed by WHO, including colistin, quinolones, and macrolides. The common use of quinolones and colistin in the study area is of particular concern as these antimicrobials constituted third line of drugs for treatment in human medicine [3]. A similar study conducted by Joosten et al. [21] reported that aminopenicillins, fluoroquinolones and tetracycline were the most frequently used antimicrobials in broilers in nine European countries and only 3 to 26% of drugs were of the “highest priority” group of drugs. In our study, ciprofloxacin and enrofloxacin (quinolones) were mostly found alone as suspension of active ingredients, while colistin and erythromycin were typically found in cocktails with other antimicrobials like trimethoprim, doxycycline, amoxicillin, sulphonamides, and oxytetracycline. Over 60% of the products contained more than one antimicrobial agent. This is similar to a previous report which found that about 60% of antimicrobial products used in small scale chicken farms in Mekong Delta of Vietnam contained multiple antimicrobial active ingredients [3]. The clinical considerations for multiple antimicrobial therapy is to cover the spectrum of potential pathogens during poly-microbial infections or in acute infections for which the responsible micro-organism or resistance profile of the pathogen is unknown [22]. Using different classes of antimicrobial drugs in combination might result in synergistic antimicrobial interactions that enhance the inhibitory effect [22]. Nonetheless, the general benefits of combination therapy compared with single or sequential administration of antimicrobials for treating bacterial infections have been difficult to conclusively demonstrate [23], and recent studies have demonstrated that combination therapy resulted in higher frequency of multi drug resistance in *Pseudomonas aeruginosa* than single drug treatment [24]. It was also observed that 5% of the farms used furazolidone even though this drug has been banned for use in Nigeria since 2017 [25]. The continued use of furazolidone illustrated the lack of compliance of poultry farmers to this regulation. A similar study reported 5% usage of banned nitrofurantoin in commercial chicken farm in Yaounde, Cameroun [14].

This study highlighted a high level of antimicrobial usage (mg/kg) per week across all the categories of farms that raised broilers and layers in the study area. As far as we were able to determine, commercial poultry feed producers do not incorporate antimicrobials in feed in Nigeria, and thus the study probably provides an overview of the total AMU in the sampled poultry farms. From the result, one can infer that farmers in the study area administer 421.5 mg (28.1 mg/kg) per chicken of antimicrobial agents to broiler chicken for a 6 weeks production period. This is even higher than the 158.2 mg per chicken of antimicrobial agents used to produce one broiler in Mekong Delta of Vietnam [26]. The high usage observed in this study could be linked to real or perceived higher prevalence of disease, the lack of government restriction and control on antimicrobial usage and inappropriate adherence to dosing intervals [27]. Qualitative studies conducted elsewhere in Nigeria and in Uganda, using questionnaire surveys, have reported a high usage of antimicrobials in poultry farms [28, 29]. A recent report used a combination of antimicrobial sales data, food animal census and countries meat consumption to project global antimicrobial use in food animals for 2017 and 2030 [6]. This study further estimated a projected increase of 11.5% from 93,309 t of active antimicrobial ingredients in 2017 to 104,079 t by 2030. In the mentioned report African countries used lower quantities of antimicrobials in 2017 compared to other continents, but the continent is expected to experience the highest increase in antimicrobial consumption (37%) by 2030. This increase is likely to be higher in Nigeria due to its increase in population [30], underlining the need to get AMU under better control in the country.

Farms that raised broilers used higher amount of antimicrobials compared to layer farms. The high usage in broilers may be attributed to the common practice of administering antimicrobials and vitamins at the beginning of production cycle. Additionally, broiler farmers have been known to use antimicrobials for growth promotion and feed efficiency [31]. Also an earlier study conducted by Filippitzi et al. [32] showed that AMU expressed as doses per unit of animal-time was highest in broiler production followed by pig and dairy. Similarly, studies conducted using various metrics to quantify antimicrobial usage in Mekong Delta in Vietnam reported high usage of antimicrobial in broiler chickens [3, 26], though this was not compared with layer birds. In some developed countries, broiler production is performed with low use of antimicrobials [33–36]. Production in such countries could form models for how to reduce AMU and subsequently AMR in LMIC, focusing on prevention of disease through biosecurity management and use of vaccines, rather than use of antimicrobials.

This study observed decreasing usage of antimicrobials as the farm sizes increased. Backyard poultry production systems with flock size less than 200, that constitute the majority of poultry industry in Nigeria [37], administered higher amounts of antimicrobials compered to larger farms. This outcome was surprising and could be due to the fact that most backyard farms do not have consulting veterinarians, lack of technical ability to administer antimicrobials correctly, lower loss tolerance capacity or a higher perception of risk of disease by household farm owners [26]. The situation analysis of AMR in Nigeria that preceded the country’s national action plan on antimicrobial resistance showed systematic misuse and over use of antibiotics in livestock production system putting local, national and global communities at risk [38].

The *Salmonella* isolates included in this study were tested for susceptibility to 11 antimicrobials [16]. We found a strong positive correlation between antimicrobial farm usages with percentage of antimicrobial resistance of the *Salmonella* isolates. Prediction of genes that conferred resistance to these antibiotics also correlated with AMU, although not at a significant level. This underscores the importance of reducing AMU as part of plans to reduce AMR, even though it remains to be demonstrated that this will lead to significant reduction of AMR for all drug types. A previous report demonstrated that the use of antimicrobial in livestock drives the evolution, prevalence, and dissemination of antimicrobial resistance in bacteria isolated from such food-producing animals [39]. Meta-analyses have found that the seven European countries with the highest use antimicrobials also had the highest levels of resistance [12]. In Japan, a significant correlation was reported between antimicrobial resistance in *E. coli* and AMU in cattle, pigs, and broiler and layer chickens [10]. In contrast, some studies do not find any relationship between AMU and AMR in livestock farms [40]. The direct relationship and significance observed between farm AMU and resistance should be interpreted with care, when extrapolating statistical significance for biological significance. However, the relationship observed provides an insight that development and spread of AMR due to imprudent usage of antimicrobial in poultry farms can occur.

Different antimicrobials do not have the same bio-availability, and they are not of equal importance with regards to weight and therapeutic activity due to difference in their potency. In the current study, we have assumed that drug presence in the target site, irrespective of the potency, influence development of resistance relatively to the weight of the chicken. Since we compare between high and low AMU at farms with aggregated resistance estimates across different antimicrobials, this may cause some inaccuracy. However, we believe the data clearly supports that magnitude of antimicrobial usage is associated with the likelihood of resistance development in gut-intestinal bacteria in a poultry farm, especially those that raised less than 200 chickens. In these farms, more antimicrobial types and higher usage in the magnitude of antimicrobials was observed compared to larger farms. Administration of cocktails of antimicrobials could subject pathogens to increased selection pressure and more likelihood of developing resistance.

## Conclusion

A wide range of antimicrobial products containing cocktails of antimicrobial active ingredients were used in commercial poultry farms in Nigeria. Relatively higher usage of antimicrobial agents per chicken per unit time was observed in broilers farms compared to chickens in layer farm, and resistance in *Salmonella* isolates from poultry farms was associated with the magnitude of antimicrobial use in farms.

## Methods

### Study area

This study was conducted in Northwest Nigeria. The area was selected because of its large geographical size, high human population density, and large poultry production as previously described [41].

### Study design

Forty-one farms were selected using a multistage technique [42] to include five categories of poultry farms (backyard, semi-commercial, small-scale, medium-scale and large-scale farms) to obtain data on AMU (Additional file 3). Typical commercial poultry farms in Nigeria are categorized into five groups based on the number of chicken raised, level of biosecurity and production output: Backyard farms (less than 200 birds), semi-commercial farms (200–999 birds), small-scale farms (1000–4999 birds), and medium-scale farms (5000–9999 birds) to large-scale farms (more than 10,000 birds). The backyard farms represent the majority of the farms sampled in this study as they constituted a large proportion of the poultry production in Nigeria [37]. An active longitudinal study design was used for 1.5 to 6 months for broilers and layers, respectively. Each selected poultry farm was paid three visits (2 and 8 weeks interval for broiler and layer farms, respectively) to obtain data about AMU and prevalence of *Salmonella* infection and degree of environmental contamination at the farm. In the first visit, written consent to participate was obtained from farmers, and they were informed on their liberty to withdraw from the study at any time. In addition, farmers were trained on how to document used antimicrobial products by putting all used sachets and other types of packet material in a plastic container and by recording the information on an AMU data sheet. For medium and large-scale farms, the AMU data sheets (Additional file 4) were further given to the consulting veterinarian to record information about product used. In the second visit, we evaluated the AMU data collected at the farm by ensuring that farmers adhered to the training provided on how to collect and register their AMU data. During the last visit, AMU data were collected and recorded for analysis. Information about antimicrobial products and their formulations including the commercial name, route of administration, antimicrobial composition and number of containers used was obtained. The Anatomical Therapeutic Chemical classification system for veterinary medicinal products (ATCvet) was used for antimicrobial drug identification [43].

### Estimation of antimicrobial usage

The formula adapted by Carrique-Mas et al. [26] with little modification was used to estimate usage in mg/kg per week (Uwc milligrams). This was obtained by dividing the multiple of the number of used antimicrobial products (Np) and amount of each active antimicrobial ingredient contained in the products (Ur milligrams) by the multiple of the length of reporting period for that farm (t weeks) and number of chickens present in the farm (Nc chickens) at the initial visit date divided by the weight (W) kg of the chicken at the end of the study period. The weight of broilers after the growth period of 6 weeks was estimated to be 2.5 kg, while that of layers after 24 weeks was 1.8 kg.

$$ Uwc=\frac{Ur\ x\  Np}{t\ x\  Nc/ Wkg} $$

### *Salmonella* status of farm and antimicrobial resistance profiles of isolates

In a cross sectional survey, carried out in parallel to the current study, pooled samples of shoe socks of fresh faecal droppings and dust samples were collected from the same 41 commercial poultry farms yielding a total of 82 samples collected to determine the prevalence and antimicrobial resistance profile of strains [41] and the reader is referred to that study for a detailed description of methods.

### Data management and statistical analysis

Data from farm AMU were entered into Microsoft Excel 2016 (Microsoft Corporation, Redmond, WA, USA) spreadsheet for descriptive statistical analysis and then exported to IBM SPSS statistic 26.0 for inferential analysis. One-way analysis of variance and student unpaired t-test was used to test for significance between AMU and categorical variables (location, type of bird and farm category). Data was exported into statistical software R for correlation and modelling using relevant installed packages (cor.test and lm function) [44]. Pearson moment correlation was used to check for correlation between usage and resistance in the isolated *Salmonella*. AMU at the farm was categorized into low and high usage based on a previous estimate of 26.4 mg usage per chicken per week (10.6 mg/kg of broilers) [25]. AMU less than 10.6 mg/kg was termed low, while usage above this cut-off value was considered as high. To investigate if usage could predict resistance, a two-step statistical approach was employed. In the first step, univariate analysis was used to check for association between resistance and variables (magnitude of usage, production type (broiler or layer) and farm category). In the second step, statistical significant predictors were selected for logistic regression. Logistic regression was used to evaluate if the total antimicrobial active ingredients (magnitude of usage) consumed per week at farm level could predict resistance found in *Salmonella* to antimicrobials used on the farm. This was done by fitting the relationship into the equation.

$$ y=a+{x}_1b+{x}_2b $$

Where, *y* (outcome) is the AMR status of farms (resistance vs non-resistance), *x* is the predictor where *x*_1_ is the AMU status of a farm (high or low, based on the cut off value described above) and *x*_2_ is the the different farm type based on flock size) and a is the intercept and b is the slope.

Ggplot2 was used to create scatter plots. Values of *p* less than 0.05 were considered statistically significant.

## Supplementary Information

**Additional file 1.** Antimicrobial compounds observed at poultry farms in Nigeria with active antimicrobials as declared by the manufacturer on product labels.

**Additional file 2 **Details of *Salmonella* isolates included in the study.

**Additional file 3.** Estimation of antimicrobial active ingredients used in commercial poultry farms. Estimation of antibiotic usage in products used in commercial farms.

**Additional file 4.** Data collection sheets used on individual farms.

## Data Availability

All data produced as part of the current study is shown in the main text and the supplementary files.

## Abbreviations

AAI: Antimicrobial active ingredients
AMU: Antimicrobial usage
AMR: Antimicrobial resistance
ARGs: Antibiotic resistance genes
LMIC: Low middle-income countries
MDR: Multidrug resistance
NAFDAC: National Agency for Food Drugs Administration and Control
NCDC: Nigerian Centre for Disease Control and Prevention
OIE: World Organization for Animal Health
WHO: World Health Organization
