# Supplementary material for: Association between antimicrobial usage and resistance in Salmonella from poultry farms in Nigeria
Source: BMC Vet Res. 2021 Jul 2;17:234. doi: 10.1186/s12917-021-02938-2 (PMC8254292; doi:10.1186/s12917-021-02938-2)
Supplement: Supplementary file 1 — Additional file 1. Antimicrobial compounds observed at poultry farms in Nigeria with active antimicrobials as declared by the manufacturer on product labels. [file 12917_2021_2938_MOESM1_ESM.docx]

**Additional file 1:** Antimicrobial compounds observed at poultry farms in Nigeria with active antimicrobials as declared by the manufacturer on product labels.

| N | Antimicrobial products ® | Antimicrobial active ingredients (AAI) | | | | | Number of AAI |
| --- | --- | --- | --- | --- | --- | --- | --- |
| 1 | Amoxy-col | Amoxycillin | Colistin |  |  |  | 2 |
| 2 | Amoxycolinor | Amoxycillin | Colistin |  |  |  | 2 |
| 3 | Amprocox | Amprolium | Sulphaquinozalone |  |  |  | 2 |
| 4 | Bidox-N | Neomycine | Doxycycline |  |  |  | 2 |
| 5 | Bruxine | Oxytetracycline |  |  |  |  | 1 |
| 6 | Cerylvet | Erythromycin | Oxytetracycline | Streptomycin | Neomycine | Colistin | 5 |
| 7 | Ciprosol 20 % | Ciprofloxacin |  |  |  |  | 1 |
| 8 | Colidox | Colistin | Doxycycline |  |  |  | 2 |
| 9 | Colizal | Tylosine | Erythromycin | Neomycine |  |  | 3 |
| 10 | Conflox 20 % | Enrofloxacin |  |  |  |  | 1 |
| 11 | Doxityl | Doxycycline | Tylosine |  |  |  | 2 |
| 12 | Doxycol | Doxycycline | Colistin |  |  |  | 2 |
| 13 | Doxy-col | Doxycycline | Colisitn |  |  |  | 2 |
| 14 | Doxy-gen | Doxycycline | Gentamicin |  |  |  | 2 |
| 15 | EST mix | Erythromicin | Sulphadiazine | Trimethoprim |  |  | 3 |
| 16 | Florecol 10 % | Florfenicol |  |  |  |  | 1 |
| 17 | Floxad 10 % | Enrofloxacin |  |  |  |  | 1 |
| 18 | Gendox | Gentamicin | Doxycycline |  |  |  | 2 |
| 19 | Gentadox | Gentamicin | Doxycycline |  |  |  | 2 |
| 20 | Gentamicin | Gentamicin |  |  |  |  | 1 |
| 21 | Gentylo | Gentamicin | Tylosin |  |  |  | 2 |
| 22 | Interseryl | Erythromicin | Oxytetracycline | Streptomicin | Colisitn |  | 4 |
| 23 | Intergendox | Gentamicin | Doxyxcycline |  |  |  | 2 |
| 24 | Kenflox 20 % | Enrofloxacin |  |  |  |  | 1 |
| 25 | Keproceryl | Colistin | Oxytetracycline | Erythromycin | Streptomycin |  | 4 |
| 26 | Megadox-N | Oxytetracycline | Neomycine |  |  |  | 2 |
| 27 | Neoceryl Plus | Oxytetracycline | Neomycine |  |  |  | 2 |
| 28 | Neodox | Neomycine | Doxycycline |  |  |  | 2 |
| 29 | Neofuramicine | Furazolidone | Oxytetracycline | Erythromycin | Streptomycin |  | 4 |
| 30 | Oxytetracycline | Oxytetracycline |  |  |  |  | 1 |
| 31 | Penprovit | Procaine Penicillin G |  |  |  |  | 1 |
| 32 | Rijoxy | Oxytetracycline |  |  |  |  | 1 |
| 33 | Sulphamix | Sulphadimidine | Sulphadiazine | Sulphathiazole |  |  | 3 |
| 34 | Samoxine | Oxytetracycline |  |  |  |  | 1 |
| 35 | Teracyclin | Oxytetracycline |  |  |  |  | 1 |
| 36 | Terramycin | Oxytetracycline |  |  |  |  | 1 |
| 37 | Tydovet | Tylosne | Doxycycline |  |  |  | 2 |
| 38 | Tylodox | Tylosine | Doxycycline |  |  |  | 2 |
| 39 | Tylo-Dox Extra | Tylosine | Doxycycline |  |  |  | 2 |
| 40 | Tylomax | Tylosine | Doxycycline |  |  |  | 2 |
| 41 | Tylovet | Tylosine | Doxycycline |  |  |  | 2 |
