# Supplementary material for: Association between antimicrobial usage and resistance in Salmonella from poultry farms in Nigeria
Source: BMC Vet Res. 2021 Jul 2;17:234. doi: 10.1186/s12917-021-02938-2 (PMC8254292; doi:10.1186/s12917-021-02938-2)
Supplement: Supplementary file 4 — Additional file 4. Data collection sheets used on individual farms. [file 12917_2021_2938_MOESM4_ESM.docx]

**Additional file 4:** Individual farm antimicrobial used on individual farms

**Farm Data**

| 1.1 | Date of Survey |  |
| --- | --- | --- |
| 1.2 | Name of farm/farm ID |  |
| 1.3 | Local Government and State |  |
| 1.4 | Number of birds |  |
| 1.5 | Farm Category | **󠆹**Backyard **󠆹󠆹**Semi commercial **󠆹󠆹**Small scale **󠆹󠆹**Medium scale **󠆹󠆹**Large scale |
| 1.6 | Period of study |  |

**Antimicrobial usage**

| S/N | Name of antimicrobial product | Amount (g or L) | Quantity | ⃰Route of administration | ⁋Method of administration | †Formulation |
| --- | --- | --- | --- | --- | --- | --- |
|  |  |  |  |  |  |  |
|  |  |  |  |  |  |  |
|  |  |  |  |  |  |  |
|  |  |  |  |  |  |  |
|  |  |  |  |  |  |  |
|  |  |  |  |  |  |  |
|  |  |  |  |  |  |  |
|  |  |  |  |  |  |  |
|  |  |  |  |  |  |  |
|  |  |  |  |  |  |  |
|  |  |  |  |  |  |  |
|  |  |  |  |  |  |  |
|  |  |  |  |  |  |  |
|  |  |  |  |  |  |  |
|  |  |  |  |  |  |  |
|  |  |  |  |  |  |  |
|  |  |  |  |  |  |  |
|  |  |  |  |  |  |  |
|  |  |  |  |  |  |  |
|  |  |  |  |  |  |  |
|  |  |  |  |  |  |  |
|  |  |  |  |  |  |  |
|  |  |  |  |  |  |  |

⃰Oral, injectable ⁋In feed, in water, injections †Powder, Suspension,
